# Molecular Docking of Phytochemicals Involved in Apoptotic Pathway and Their Interactions with *CASP3*, *CASP9*, and *BAX* in HepG2 Cell Line

**DOI:** 10.3390/plants15121822

**Published:** 2026-06-12

**Authors:** Madiha Younas, Muhammad Zubair, Muhammad Yousaf Shani, Samia Ahmad, Iqra Arshad, Wacław Jarecki, Muhammad Azmat, Ghulam Farid, Muhammad Yasin Ashraf, Lanlan Zhu

**Affiliations:** 1 College of Agricultural Engineering and Food Science, Shandong University of Technology, Zibo 255000, China; madihayounas164@gmail.com; 2 Department of Bioinformatics and Biotechnology, Government College University Faisalabad, Faisalabad 38000, Pakistan; muhammadzubair1722@gmail.com; 3 Nuclear Institute for Agriculture and Biology, College (NIAB-C), Pakistan Institutes of Engineering and Applied Science (PIEAS), Islamabad 45650, Pakistan; mmyousafshani@gmail.com (M.Y.S.); robinaaziz347@gmail.com (I.A.); fareedniab@yahoo.com (G.F.); niabmyashraf@gmail.com (M.Y.A.); 4 Institute of Molecular Biology and Biotechnology, The University of Lahore, Lahore 54000, Pakistan; samiaahmaduol436@gmail.com (S.A.); muhammadazmat557@gmail.com (M.A.); 5 Department of Crop Production, University of Rzeszów, Zelwerowicza 4, 35-601 Rzeszów, Poland; wjarecki@ur.edu.pl; 6 Department of Molecular Biology and Genetics, Faculty of Engineering and Natural Sciences, Biruni University, 34015 Istanbul, Türkiye

**Keywords:** AgNPs, *CASP3*, bioactive compounds, docking

## Abstract

As liver cancer is a leading cause of death all over the world, there is a need to explore new therapeutic strategies. This study presents an in silico analysis of the genes Caspase_3_ (*CASP_3_*), Caspase_9_ (*CASP_9_*), and *BCL-2-associated X protein* (*BAX*) in liver cancer cells to evaluate the apoptosis profile following exposure to green-synthesized plant extract. We assessed the modulatory effects of phytochemicals on the apoptotic pathway by means of bioinformatics tools and a publicly available gene expression dataset. Our findings revealed the possible mechanistic basis of the pro-apoptotic activity observed in vitro, utilizing a structure-based molecular docking method. The biologically synthesized AgNPs at a concentration of 50 µg/mL induced an approximately 4-fold increase in the mRNA expression levels of *CASP3*, *CASP9*, and *BAX* compared with chemically synthesized AgNPs, as determined by qPCR. Rutin was the compound with the highest binding affinities toward all three proteins, with ΔG values of −9.3 kcal/mol (Caspase_3_), −9.1 kcal/mol (Caspase_9_), and −9.0 kcal/mol (*BAX*). These findings offer new insights about the molecular mechanisms that support the cytotoxicity of phytochemicals, and simultaneously highlight the potential of green nanotechnology for the development of therapeutic strategies for liver cancer.

## 1. Introduction

Hepatocellular carcinoma (HCC) is the most common form of primary liver cancer (PLC) and is considered one of the most aggressive malignancies worldwide [1,2]. At present, it is one of the five leading causes of cancer-related mortality worldwide, with the five-year survival rate for most patients remaining below 20% [3,4]. Epidemiological studies predict that the global burden of liver cancer may reach approximately one million cases annually in the coming years [5]. More than 80% of HCC cases occur in low- and middle-income countries, where healthcare resources and early diagnostic facilities are limited [6,7].

The disease is particularly prevalent in Africa and Asia [8]. Despite advancements in therapeutic strategies, the prognosis of HCC remains poor because the disease is often diagnosed at advanced stages, and conventional treatments show limited long-term effectiveness [9]. Therefore, the development of safer and more effective therapeutic approaches for HCC management remains a major challenge in cancer research [10]. Apoptosis, or “programmed cell death,” is a highly regulated biological process for eliminating damaged, aged, or abnormal cells while maintaining tissue homeostasis [11]. In cancer cells, apoptotic signaling pathways are frequently dysregulated, enabling uncontrolled proliferation and resistance to therapy. Consequently, restoration or induction of apoptosis has emerged as a promising strategy for anticancer drug development [12]. However, currently available therapeutic approaches for HCC, including chemotherapy, radiotherapy, surgical resection, and liver transplantation, are associated with limitations such as drug resistance, adverse side effects, tumor recurrence, and long-term immunosuppression [13,14].

In recent years, nanotechnology has attracted significant attention in cancer research because of its potential applications in targeted drug delivery, imaging, diagnosis, and therapy [4,15]. Nanomaterials possess unique physicochemical properties that can improve the bioavailability and therapeutic efficiency of anticancer agents. Among the various approaches, green synthesis of nanoparticles using plant extracts has gained increasing importance because it is environmentally friendly, cost-effective, and biologically safe [16]. Plant-mediated synthesis avoids the use of toxic chemicals through the utilization of naturally occurring phytochemicals as reducing, stabilizing, and capping agents during nanoparticle formation. Researchers have leveraged the unique properties of nanomaterials, whether nanocrystalline or nanostructured, to facilitate cancer diagnosis and therapy [17,18].

Phytochemicals are naturally occurring bioactive compounds produced by plants for defense and survival. These compounds, including flavonoids, phenolic acids, terpenoids, alkaloids, and tannins, possess diverse pharmacological activities such as antioxidant, anti-inflammatory, antimicrobial, and anticancer properties [19]. Several phytochemicals have been reported to regulate apoptosis by modulating key signaling pathways associated with cancer progression [20,21]. Due to these biological activities, phytochemicals have become important candidates in the development of novel anticancer therapeutics and nanoparticle-based drug delivery systems [22,23].

Previous phytochemical analyses of *Trianthema portulacastrum* and *Chenopodium quinoa* have identified the presence of rutin, ferulic acid, aleuritolic acid, and several flavonoid derivatives [24,25]. These bioactive compounds are known to participate in apoptosis-related pathways and may contribute to the anticancer activity of plant-derived nanoparticles. Therefore, four representative compounds, including rutin, ferulic acid, aleuritolic acid, and a basic flavonoid structure, were selected for molecular docking against key apoptotic proteins (*BAX*, *CASP_3_*, and *CASP_9_*) to evaluate their binding interactions and therapeutic potential, based on their previously reported pro-apoptotic and anticancer activities [26,27].

Computational approaches such as molecular docking have become valuable tools in modern drug discovery because they enable the prediction of ligand–protein interactions, binding affinities, and possible mechanisms of action [28,29]. Bioactive substances from medicinal plants are not only responsible for plant defense but are also useful in biomedical applications, especially in cancer treatment by influencing the pathways that control apoptosis [30]. During the green synthesis of nanoparticles, plant-derived bioactive compounds act as reducing, capping, and stabilizing agents, facilitating eco-friendly nanoparticle formation and enhancing their biological activity [31]. *Trianthema portulacastrum* and *Chenopodium quinoa* were selected in the present study because both species are rich sources of phenolics, flavonoids, and terpenoids with strong antioxidant and anticancer potential [32,33,34].

Combined with experimental validation, these in silico techniques can accelerate the identification of biologically active compounds and therapeutic targets. In our previous studies [35,36], the cytotoxic effects of green and chemically synthesized nanoparticles were evaluated using MTT assays. Building upon these findings, the present study further investigates the apoptotic mechanisms underlying nanoparticle-induced cytotoxicity in HepG2 cells through Reverse Transcription Quantitative Polymerase Chain Reaction (RT-qPCR) analysis of apoptosis-related genes (*CASP_3_*, *CASP_9_*, and *BAX*) and molecular docking studies of selected phytochemicals. The overall objective of this work was to explore the mechanistic relationship between phytochemical-mediated nanoparticle synthesis and apoptosis induction in HCC cells using both experimental and computational approaches.

## 2. Results

### 2.1. Relative Gene Expression Analysis of Apoptotic Markers in Green and Chemically Synthesized Nanoparticles

**Figure 1:**
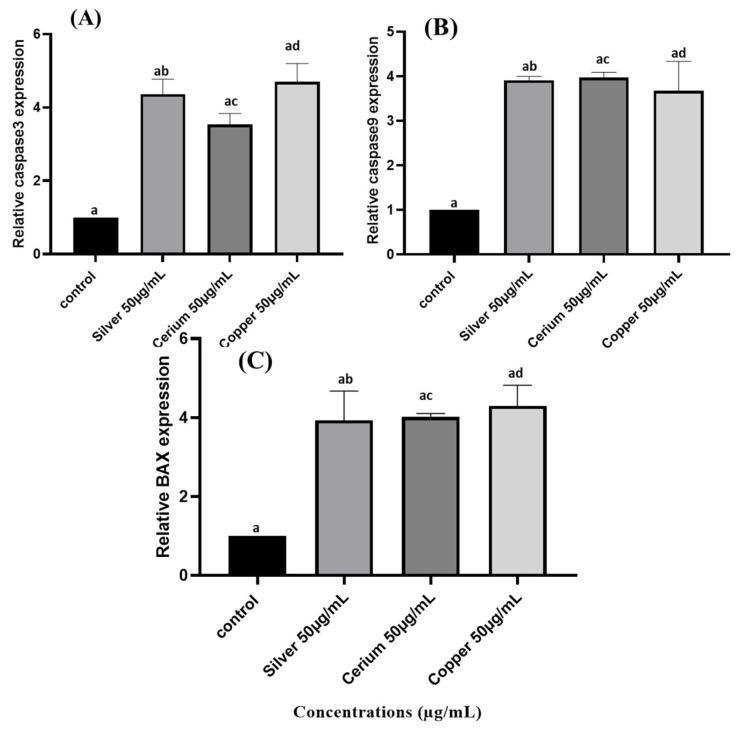
Relative expression levels of the apoptotic genes (**A**) *CASP_3_*, (**B**) *CASP_9_*, and (**C**) *BAX* in HepG2 cells treated with green-synthesized nanoparticles at a concentration of 50 µg/mL. Data are presented as mean ± SE (*n* = 3). Statistical significance was determined using Tukey’s multiple comparisons test (*p* ≤ 0.05). Different letters above bars indicate statistically significant differences, whereas bars sharing the same letter are not significantly different.

**Figure 2:**
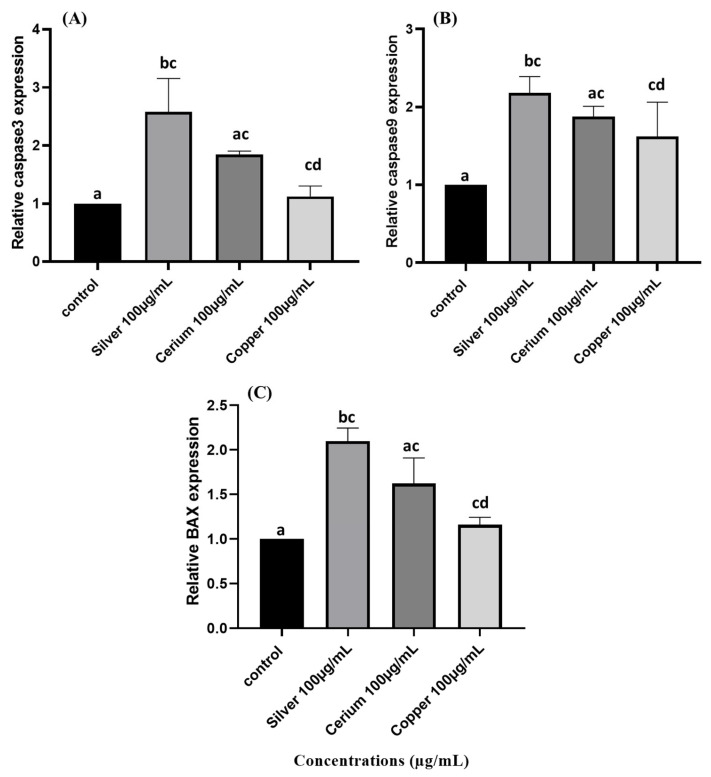
Relative expression levels of the apoptotic genes (**A**) *CASP_3_*, (**B**) *CASP_9_*, and (**C**) *BAX* in HepG2 cells treated with chemically synthesized nanoparticles at a concentration of 100 µg/mL. Data are presented as mean ± SE (*n* = 3). Statistical significance was determined using Tukey’s multiple comparisons test (*p* ≤ 0.05). Different letters above bars indicate statistically significant differences, whereas bars sharing the same letter are not significantly different.

Our previous work demonstrated that biogenic nanoparticles (Ag, Ce, and Cu) synthesized from *T. portulacastrum* and *C. quinoa* extracts induced significant dose-dependent cytotoxicity in HepG2 cells. Notably, certain formulations, like Ag-B and Ce-AB, achieved over 90% inhibition at 50 µg/mL in our previous studies [35,36], where the cytotoxicity was largely attributed to the triggering of oxidative stress. This established model of nanoparticle-induced oxidative stress provides a direct foundation for this study. The expression levels of the apoptosis-related genes *CASP_3_*, *CASP_9_*, and *BAX* were significantly elevated in HepG2 cells treated with various nanomaterials compared with untreated controls. Among the concentrations evaluated, treatment with 50 µg/mL of green-synthesized nanoparticles produced the highest induction of apoptotic gene expression. Statistically significant increases for these genes were found when increasing the dose of nanoparticles further (particularly in silver and copper), as shown in Figure 1 and Figure 2. Furthermore, the expression levels observed at 50 µg/mL of green-synthesized nanoparticles were significantly higher than those of the untreated control group (*p* ≤ 0.05), as indicated by Tukey’s multiple comparison test and the different letter groupings shown in Figure 1.

### 2.2. Molecular Docking Insights into Ligand–Apoptotic Protein Interactions

The use of AutoDock Vina (v1.5.6) for docking simulations yielded a summary of the compounds’ binding free energies (ΔG, kcal/mol) with each target as shown in Table 1. Rutin was the compound with the highest binding affinity toward all three proteins, with ΔG values of −9.3 kcal/mol (Caspase_3_), −9.1 kcal/mol (Caspase_9_), and −9.0 kcal/mol (*BAX*). The highly negative ΔG values mean that the interactions are stable and thermodynamically favorable, and so rutin is likely to be very effective in accessing either the active or the regulatory sites of apoptotic proteins. On the other hand, ferulic acid’s affinities ranged from −7.2 to −6.8 kcal/mol and were therefore much weaker, which means that the targets are not very flexible in their conformations. Aleuritolic acid and flavonoid were placed in the middle, indicating their compatibility with biological systems was more than the weak interactors, but less than that of rutin.

**Figure 3:**
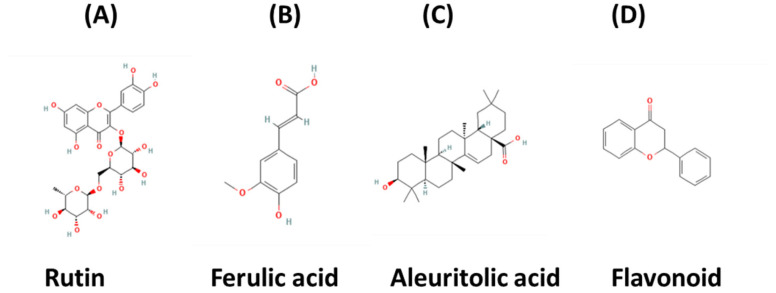
Chemical structures of the selected phytochemicals used in molecular docking analysis, including (**A**) Rutin, (**B**) Ferulic acid, (**C**) Aleuritolic acid, and (**D**) basic Flavonoid.

The compounds chosen are examples of the main phytochemical groups found in plants from which nanoparticles were synthesized (Figure 3). A representative flavonoid was also included, as flavonoids are the most abundant class in the majority of plant species. All four compounds (rutin, ferulic acid, aleuritic acid, and flavonoids) were selected because they were reportedly present at high levels, have diverse structures, and were reported to be involved in apoptosis-related pathways, thereby ensuring the biological relevance of the molecular docking studies.

**Figure 4:**
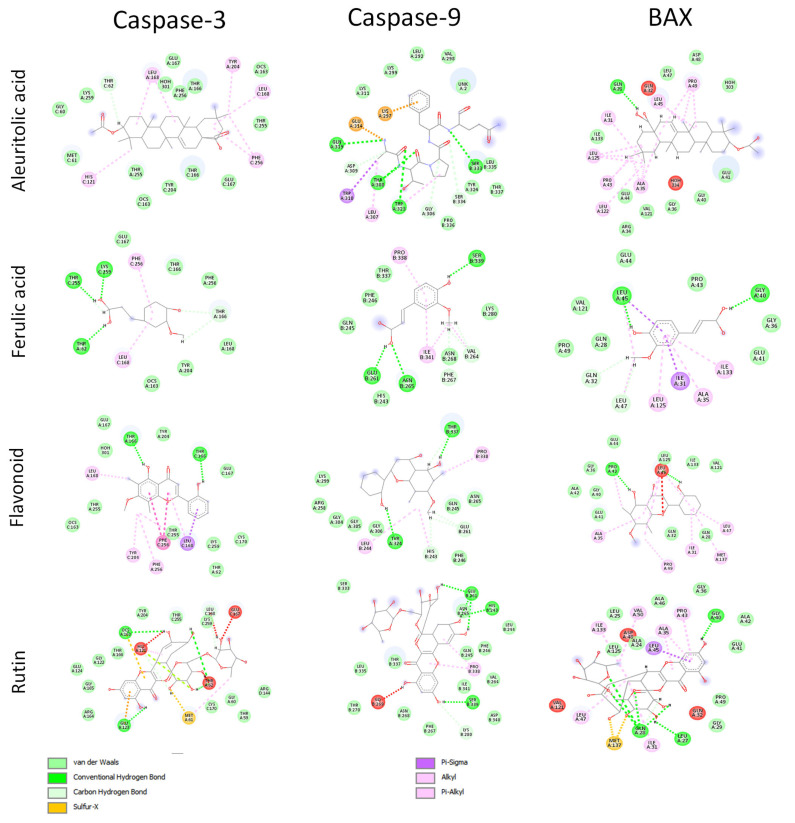
Two-dimensional interaction profiles of aleuritolic acid, ferulic acid, basic flavonoid structure, and rutin docked with *CASP_3_*, *CASP_9_*, and *BAX* proteins, generated using Discovery Studio Visualizer. Alkyl and Pi-Alkyl interactions represent hydrophobic interaction subtypes and are distinguished by their respective labels in the figure legend.

**Figure 5:**
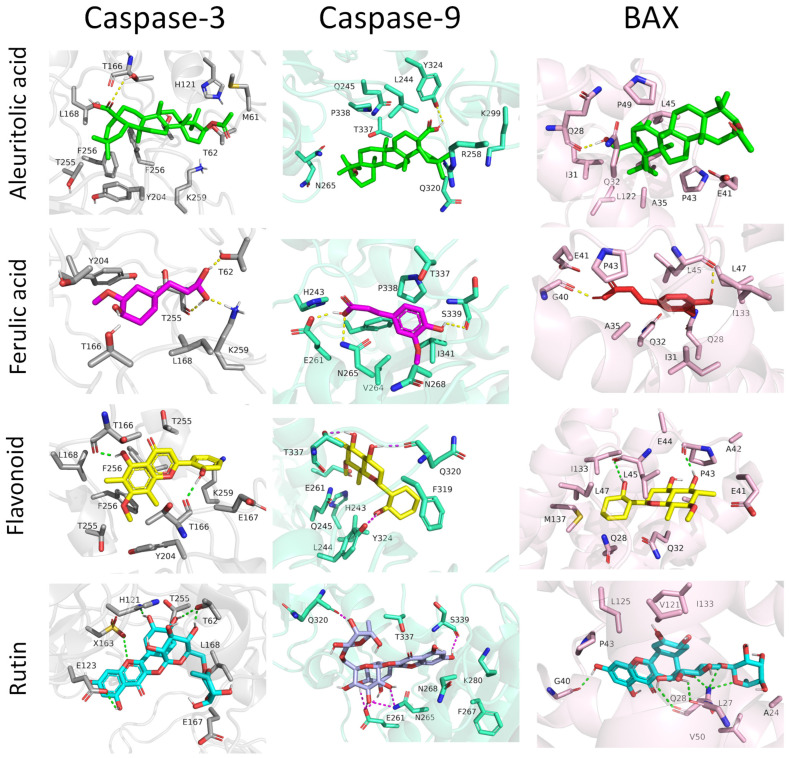
Three-dimensional binding conformations of aleuritolic acid, ferulic acid, basic flavonoid structure, and rutin with *CASP3*, *CASP_9_*, and *BAX* proteins, visualized using PyMOL.

The Rutin–BAX complex showed numerous stabilizing non-covalent interactions in the pseudo-3D representation (Figure 4). Hydrogen bonding and hydrophobic interactions were observed between rutin and several amino acid residues located within the active region of BAX, supporting stable ligand–protein interactions and a favorable binding orientation. The mentioned residues are situated close to the BAX activation loop, which is the area that is the most critical determinant of the conformational transition from an inactive to an active pro-apoptotic state. The accompanying 3D model (Figure 5) showed that rutin was deeply embedded in the hydrophobic BAX pocket, giving strong surface complementarity and a favorable steric fit, which are the hallmarks of high-affinity binding.

The Rutin–Caspase_3_ and Rutin–Caspase_9_ complexes displayed the same interaction profiles. Rutin was located in the catalytic domains of both enzymes, creating hydrogen bonds with the residues in the active site, which may contribute to enzyme stabilization and activation. Since Caspase*_9_* is the upstream initiator that activates Caspase*_3_*, this dual interaction pattern indicates that rutin may influence apoptosis at both the initiation and execution points, thereby amplifying the apoptotic signal.

Data from RT-qPCR analysis showed significant upregulation of *CASP_3_*, *CASP_9_*, and *BAX* genes in HepG2 cells treated with green-synthesized nanoparticles, confirming the in silico observations. This correlation between computational and experimental findings supports a dual mechanistic model for the apoptosis mediated by nanoparticles [37].

The nanoparticles serve as efficient nanocarriers that improve intracellular accumulation and hence the bioavailability of phytochemicals. Phytochemicals, particularly rutin, have a direct effect on apoptotic proteins and, therefore, activate programmed cell death pathways. It is likely that the combination of mechanisms accounts for the massive apoptosis of cells treated with nanoparticle-encapsulated phytochemicals compared with free phytochemicals. The high docking affinities of rutin to Caspase_3_, Caspase_9_, and *BAX*, along with the multiple bonding interactions and spatial fitting, suggest that rutin is able to activate both mitochondria-dependent and caspase-dependent apoptotic pathways simultaneously. The intermediate binding affinities detected for aleuritolic acid and the representative flavonoid imply possible co-modulatory actions, where these compounds may work together with rutin to boost the overall apoptotic response.

The numerous favorable binding energy values, residue-level interactions, and the correlation with gene expression trends support the concept that rutin has multi-functional properties as an active agent capable of inducing apoptosis. Combining both the docking results from computational modeling and experimentally derived information provides a logical explanation for increased apoptosis in HepG2 cells exposed to nanoparticles synthesized using the green synthesized nanoparticles. Therefore, rutin could be used as a lead molecule in developing anticancer therapies mediated by nanoparticles. This work also demonstrates the validity of designing targeted nanopharmaceuticals for the treatment of HCC based on rational design [38]. Therefore, this study does not imply that nanoparticles have been produced solely in the presence of rutin. Rather, based upon its ability to bind strongly, its beneficial interaction characteristics, and its compatibility with systems where plant-mediated synthesis of nanoparticles takes place, we propose that rutin may be an active ligand for nanoparticle synthesis.

## 3. Discussion

Our previous investigations demonstrated that biogenic nanoparticles (Ag, Ce, and Cu) synthesized from *Trianthema portulacastrum* and *Chenopodium quinoa* extracts induced significant dose-dependent cytotoxicity in HepG2 cells. Notably, certain formulations, like Ag-B and Ce-AB, achieved over 90% inhibition at 50 µg/mL, as reported in [35,36]. Earlier studies attributed this cytotoxic activity primarily to nanoparticle-induced oxidative stress, which provided the mechanistic basis for the present investigation into apoptosis-related molecular responses [39].

In the current study, expression analysis revealed significant upregulation of the pro-apoptotic genes *BAX* (a pivotal regulator in the extrinsic apoptosis pathway), *CASP3* (the cell-death executor in both pathways), and *CASP_9_* (the intracellular apoptosis cascade starter) in nanoparticle-treated HepG2 cells compared with untreated controls. These genes are key regulators of programmed cell death, with *CASP_9_* functioning as an initiator of the intrinsic apoptotic pathway, *CASP_3_* acting as a major executioner caspase, and *BAX* promoting mitochondrial membrane permeabilization. Their coordinated regulation suggests activation of apoptotic signaling pathways following nanoparticle exposure and highlights apoptosis as a major mechanism underlying the observed suppression of cancer cell proliferation.

In addition, gene and protein expression levels were extensively analyzed in HepG2 cells following treatment with nanoparticles. The results corroborated the RT-qPCR findings and indicated activation of the apoptosis-related cell death mechanism through enhanced expression of key apoptosis markers [40]. This suggests that nanoparticles can effectively promote the death of cancer cells by inducing apoptosis via both intrinsic and extrinsic pathways. The observed upregulation at both the gene and protein levels underscores the involvement of apoptosis as a key mechanism underlying the anti-tumor effects of nanoparticle treatments on hepatocellular carcinoma cells [41].

Among the tested concentrations, 50 µg/mL of green-synthesized nanoparticles elicited the strongest biological response, resulting in approximately 4-fold increases in *CASP_3_* and *CASP_9_* expression and a 3.5-fold increase in BAX expression. These findings are consistent with previous studies demonstrating that plant-derived nanoparticles induce apoptosis through modulation of mitochondrial and caspase-dependent signaling pathways [42,43]. Increased expression of these apoptotic markers has frequently been associated with caspase activation, mitochondrial dysfunction, and subsequent cancer cell death in HepG2 and other cancer models [44,45]. Therefore, the observed upregulation of apoptosis-related genes indicates activation of programmed cell death pathways following nanoparticle treatment and further supports the pro-apoptotic activity of the synthesized nanoparticles [46,47].

It is important to recognize that elevated transcript levels do not necessarily translate into corresponding increases in protein abundance, as gene expression is subject to extensive post-transcriptional and post-translational regulation. Thus, the enhanced expression of *CASP_3_*, *CASP_9_*, and BAX should be regarded as evidence of apoptotic pathway activation rather than direct confirmation of protein functionality. Additional protein-level investigations and enzymatic activity assays would strengthen the mechanistic interpretation of the present findings [44].

To further elucidate the molecular basis of apoptosis induction, structure-based molecular docking studies were performed using four major phytoconstituents identified in the plant extracts; namely, aleuritolic acid, ferulic acid, flavonoids, and rutin. These compounds were evaluated against three key apoptotic regulators: Caspase*_3_*, Caspase_9_, and *BAX*. Caspase_9_ acts as an initiator of the intrinsic mitochondrial apoptotic pathway, Caspase_3_ functions as the primary executioner protease, and BAX facilitates mitochondrial outer membrane permeabilization and cytochrome-c release [45]. Docking analyses demonstrated favorable interactions between the selected phytochemicals and all three target proteins, suggesting their potential involvement in apoptosis regulation.

Among the investigated compounds, rutin displayed the strongest binding affinities and the most favorable interaction patterns, indicating a high degree of structural compatibility with the apoptotic targets. This observation is consistent with previous reports describing rutin as a multifunctional flavonoid capable of modulating apoptosis-related signaling through strong interactions with regulatory proteins [48,49]. Similar findings have been reported in various cancer models, where rutin and other polyphenolic compounds promoted apoptosis and suppressed tumor progression by targeting proteins involved in oxidative stress and programmed cell death [50,51]. The superior binding performance of rutin may be attributed to its multiple hydroxyl groups and aromatic rings, which facilitate extensive hydrogen bonding and π–π stacking interactions within protein-binding sites.

The phytochemicals evaluated in this study represent major bioactive constituents of the plant extracts used for nanoparticle synthesis. Because the nanoparticles were generated from complex botanical extracts rather than a single purified compound, the observed biological effects are likely attributable to synergistic interactions among multiple phytoconstituents together with nanoparticle-mediated cellular delivery. The favorable docking interactions, coupled with the marked upregulation of apoptotic genes, suggest that these compounds collectively contribute to the activation of apoptosis-related signaling pathways in HepG2 cells. The concordance between the computational predictions and gene-expression data therefore supports a cooperative role of plant-derived phytochemicals and nanoparticle-mediated mechanisms in promoting apoptosis.

Although molecular docking provides valuable insight into potential ligand–protein interactions, it remains a predictive and inherently static computational approach. Consequently, future studies involving molecular dynamics simulations, protein expression analyses, and functional validation assays are required to verify the stability, biological relevance, and mechanistic significance of the predicted interactions. Such investigations will provide a more comprehensive understanding of the therapeutic potential of phytochemical-mediated nanoparticles as apoptosis-inducing agents against liver cancer cells.

## 4. Materials and Methods

### 4.1. Cell Line Culture

**Figure 6:**
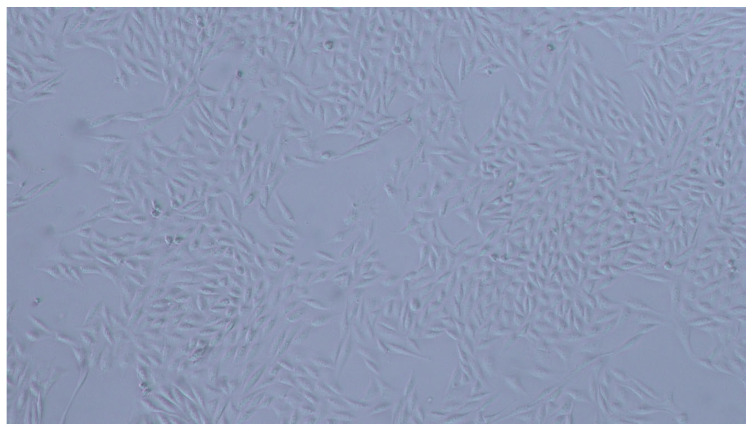
Human cancer cell lines HepG2 cultured in DMEM maintained at 37 °C in a humid atmosphere with 5% CO_2_.

*T. portulacastrum* and *C. quinoa* were chosen to synthesize nanoparticles due to their rich phytochemicals, specifically flavonoids, phenolic acids, and triterpenoids, as well as their previously documented anticancer activities. In addition to being utilized as effective reducing and stabilizing agents in green nanoparticle synthesis, they may be useful as biological agents [52]. These plant extracts and nanoparticles were first synthesized and tested for their cytotoxic effects on HepG2 cells as part of our previous study [35]. Specifically, we investigated cerium oxide (CeO_2_), silver oxide (AgO_2_), and copper oxide (CuO) nanomaterials that were made using both a chemical precipitation method as well as a green precipitation method. We utilized these materials in this study for the purposes of conducting apoptosis-related gene expression analyses and performing molecular docking experiments. The present study builds directly on our previous work by extending the investigation toward mechanistic understanding through gene expression analysis and molecular docking. The cells were maintained in a 75 mL cell culture flask with 10 mL Dulbecco’s modified Eagle’s medium (DMEM) supplemented with 10% fetal bovine serum (FBS) and 1% 100X L-Glutamine to promote cell proliferation (Figure 6). The cell line was incubated at 37 °C for 24 h in a humid environment with 5% CO_2_. After 2–3 days, medium was replaced with fresh medium. Trypsin (2–5 mL) was applied to detach the cells from the surface. When the live cells detached from the flask’s surface, 10 mL of fresh DMEM was added and cell suspension was transferred to new culture flasks, allowing cells to reattach and divide further. These cells were employed in subsequent experiments and analyses.

### 4.2. Potential of Anticancer Activity

**Figure 7:**
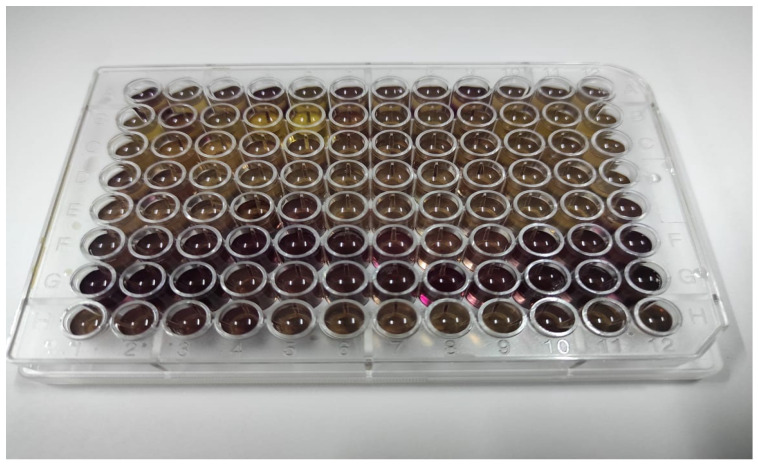
The 96-well plate used to determine the anticancer activity.

The HepG2 cell line, derived from human liver carcinoma, is widely utilized in research to evaluate potential anticancer agents. In this study, HepG2 cells were cultured in 96-well microplates, with each well containing 2 × 10^4^ cells (Figure 7). The cells were exposed to three different types of nanoparticles (NPs)—namely, silver oxide (AgO_2_), cerium oxide (CeO_2_), and copper oxide (CuO_2_)—which were synthesized through a green and chemical precipitation method [35,36]. These nanoparticles were prepared at various concentrations (10 µg/mL, 50 µg/mL, and 100 µg/mL) and dissolved in phosphate-buffered saline (PBS) to ensure uniform dispersion. A control group was maintained without any nanoparticle treatment to serve as a negative control for comparison. These concentrations were chosen due to the preliminary results of a dose–response experiment in our previous study, in which we demonstrated that, at those concentrations, there were reliable and measurable cytotoxic effects in HepG2 cells. In addition, the use of multiple concentrations (10, 50, and 100 µg/mL) allowed for comparison among them. Finally, 50 µg/mL was determined to be the most suitable concentration for downstream gene expression analysis.

Following a 24 h incubation period to allow the nanoparticles to interact with the cells, 10 μL of MTT reagent (3-(4,5-dimethylthiazol-2-yl)-2,5-diphenyltetrazolium bromide) at a final concentration of 5 mg/mL was added to each well. The plates were then incubated for an additional 4 h to facilitate the reduction of MTT by metabolically active cells, resulting in the formation of insoluble formazan crystals. Crystals were dissolved by adding 150 μL DMSO, and the absorbance was measured using a microplate reader at 570 nm.

The following formula was used to determine the proportion of cell proliferation reduction to evaluate the cytotoxic effects of the nanoparticles [37]:

$$Cytotoxicity=\frac{Ac-As}{Ac}$$

where *Ac* stands for the absorption rate of the control group (untreated cells), and *As* denotes the absorbance of the sample (cells treated with nanoparticles). The degree to which each nanoparticle and concentration suppresses the growth of HepG2 cells is shown by this quantitative method, suggesting that they may have anticancer properties. We have provided findings related to cytotoxicity responses and apoptosis from experiments performed using the same methods as those used for this research in Figure 1 and Figure 2. These findings were also verified through our prior research on cytotoxicity [35,36].

### 4.3. Gene Expression Analysis

The primers used in this investigation were created with the NCBI Primer-BLAST (Primer3 version 2.5.0) tool, a dependable web-based tool that enables the construction of particular primer pairs that are suited for the targeted sequences of interest. For real-time PCR analysis, a primer pair was selected for its ability to produce DNA fragments between 150 and 200 base pairs (bp)—the ideal size range for amplification efficiency and specificity, enabling precise and sensitive detection of the target genes. The gene names and corresponding primer sequences used in this study are listed in Table 2. These primers were carefully validated to ensure their specificity and efficiency, contributing to the robustness of the experimental results. Additionally, GAPDH was used as the internal housekeeping gene for normalization of relative gene expression data.

### 4.4. RNA Extraction from Cells

**Figure 8:**
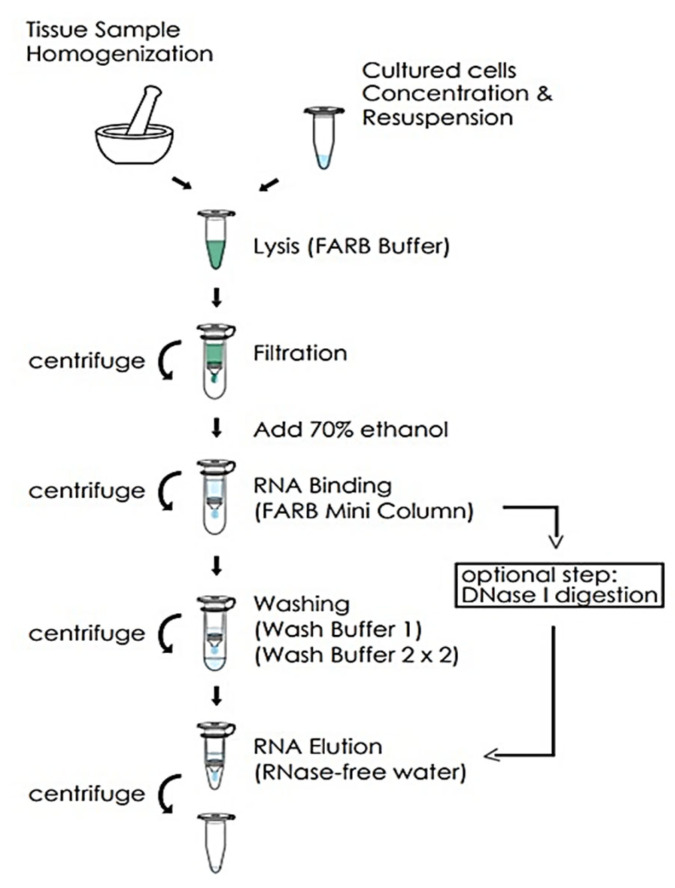
RNA extraction was carried out following the manufacturer’s instructions. Briefly, cells were lysed using Trizol reagent and then homogenized through pipetting.

Highly pure mRNA was extracted from HepG2 cells using the FavorPrep Tissue Total RNA Mini Kit, following the manufacturer’s instructions, which included lysing the cells with Trizol reagent and homogenizing them by pipetting (Figure 8). Briefly, cells were lysed using Trizol reagent and then homogenized through pipetting. The RNA obtained after extraction was subsequently utilized for downstream gene expression analysis of *CASP_3_*, *CASP_9_*, and *BAX* in treated HepG2 cells.

### 4.5. DNA Synthesis

Thermo Scientific’s RevertAid First Strand cDNA synthesis (Thermo Fisher Scientific, Vilnius, Lithuania) kit was used to create the cDNA in two stages. DNase was used to purify the RNA in the first step, and cDNA was made in the second step in accordance with the specified procedure. A sterile nuclease-free microcentrifuge tube was filled with 1 µL of 15 pmol/µL gene-specific primer after the kit’s components had been thawed, mixed, and briefly centrifuged. The tube was then incubated for 5 min at 65 °C and cooled on ice for 60 s. A reaction mixture with 4 µL of 5X reaction buffer, 20 units of RNase inhibitor (1 µL), 2 µL of 10 mM dNTPs mix, and 200 units of RevertAid M-MuLV RT (1 µL) was added to the template PCR tube. After gently mixing the tube, it was incubated for 60 min at 42 °C and then for a further five minutes at 70 °C. The cDNA that was synthesized was kept at −20 °C.

### 4.6. Rt-qPCR

Master mix for the PCR (50 µL final volume) was prepared in a sterile microcentrifuge tube by means of the components listed in Table 3. The CFX96 RT-PCR Detection System (Bio-Rad) was used for the quantitative PCR (qPCR), and the fluorescent detection reagent was Maxima™ SYBR Green Master Mix (2X). The first step in the reaction setup was the careful addition of cDNA into the bottom of a flat-cap PCR tube, then the master mix was added for mixing and reaction consistency. The polymerase was started up, and the full denaturation of the DNA template was guaranteed by the PCR amplification under cycling conditions, starting with a preliminary denaturation at 50 °C for 10 min. The DNA strands were then separated by subjecting them to 30 s of denaturation at 95 °C. For primers to bind to their matching sequences, the annealing phase took place for one minute at 55 °C. The next stage was extension, which involved one minute at 72 °C, to support the DNA synthesis process of the polymerase enzyme. All the steps (denaturation, annealing, and extension) were repeated 40 times for efficient amplification of the target sequences. The last step, elongation, was performed for three minutes at 72 °C to ensure that all amplifications were successful.

To check the characteristics of the PCR product, curve analysis of the product was performed after the completion of the amplification cycle. During the process, the temperature increased slowly from 65 °C to 95 °C within a 2 s interval at each cycle, taking those 2 s for each increment. These steps eased the detection of the melting points of amplifications and assisted in determining the combination of primer-dimer or any non-specific byproducts. These melting curve data were utilized to enhance the achievement of heat of each primer pair, so confirming the precise and accurate results of amplification. This standard process allowed for the quantification of target nucleic acid by ensuring great sensitivity and specificity, thus providing the precise data for following experiments.

### 4.7. Molecular Docking

The main aim of molecular docking simulations was to determine the binding orientation, estimate the binding affinities, and identify possible molecular interactions between the selected bioactive phytochemicals and the apoptosis-regulating target proteins Caspase_3_, Caspase_9_, and BAX. The target proteins’ high-resolution crystallographic structures were obtained from the RCSB Protein Data Bank (https://www.rcsb.org/) (accessed on 12 June 2025), while the aleuritolic acid, ferulic acid, flavonoid, and rutin chemical structures were sourced in SDF format from the PubChem compound repository.

The ligand structures underwent energy minimization utilizing the MMFF94 force field through Open Babel to achieve optimized conformers. Protein preparation, including deletion of crystallographic water molecules, addition of polar hydrogens, and computation of Gasteiger charges, was executed using AutoDockTools v1.5.6. Docking simulations were performed with AutoDock Vina, employing a grid box precisely centered on the known active or catalytic sites of each target protein, as defined by co-crystallized ligand or literature-reported residues. Each docking run yielded multiple binding poses, from which the most energetically favorable conformation (lowest binding free energy, ΔG in kcal/mol) was selected for further analysis. Flavonoids are naturally occurring compounds found in many foods and plants, including *Trianthema portulacastrum* and *Chenopodium quinoa*. To maintain consistency with the phytochemical background of these two plants, the basic flavonoid backbone was included in the molecular docking analysis. It is important to clarify that only the general flavonoid skeletal framework was used in this study, rather than a specific naturally occurring flavonoid derivative or a representative substituted flavonoid compound. This represents a limitation of the docking approach because naturally occurring flavonoids contain diverse functional group substitutions, and the presence, number, and position of these functional groups may significantly influence ligand–protein interactions, binding affinity, molecular orientation, and docking outcomes. Therefore, the docking results for the basic flavonoid structure should be interpreted as a general structural indication of possible flavonoid–protein interactions. Further studies using specific flavonoid derivatives are required to provide more detailed and biologically relevant docking interpretations.

### 4.8. Statistical Analysis

A comparison was made of the differences between the apoptotic gene expressions utilizing an unpaired Tukey’s multiple comparison test in GraphPad Prism (11.0.2). The experiments were carried out in triplicate (*n* = 3). Data are shown as mean ± standard deviation, with *p*-value < 0.05 determined to be statistically significant. Post-docking assessment was conducted via visualization and interaction profiling using PyMOL v2.5 and Discovery Studio Visualizer, which allowed for the identification of hydrogen bonding, hydrophobic interaction, and electrostatic contact present within the protein–ligand complex.

## 5. Conclusions

New therapeutic approaches are required for liver cancer, which continues to be a major cause of cancer-related death globally. In this work, the expression profiles of genes linked to apoptosis (*CASP_3_*, *CASP_9_*, and *BAX*) in liver cancer cells treated with green-synthesized nanoparticles synthesized using plant extract and a chemical method were analyzed in silico. Using bioinformatics tools and publicly available gene expression datasets, we evaluated the modulatory effects of these phytochemicals on apoptotic pathways. In a similar context, our results provide insight into the possible mechanisms underlying the pro-apoptotic activity that was observed in vitro. A structure-based molecular docking method was used to assess the binding of four active plant constituents (aleuritolic acid, ferulic acid, a representative flavonoid, and rutin) with three main apoptotic regulators: Caspase_3_, Caspase_9_, and *BAX*. The findings provided insight into molecular pathways underlying the cytotoxic effects of the plant extracts and highlighted the potential of green nanotechnology in the development of novel therapeutic strategies for the management of liver cancer. Further studies are needed to validate the computational predictions and to further assess the clinical implications. The gene expression data obtained through RT-qPCR matched the computational predictions very well, as it marked the upregulation of *CASP_3_*, *CASP_9_*, and *BAX*, particularly in the cells treated with green-synthesized nanoparticles. In silico binding data and experimental gene expression analysis suggest a dual mechanism where the nanoparticles might not only deliver the intracellular signals for apoptosis, but they might also facilitate interactions between ligands and proteins that directly trigger apoptotic cascades, thus activating apoptosis. IN this context, rutin emerges as a promising phytochemical candidate for modulating apoptotic signaling in hepatocellular carcinoma (HCC), thus strengthening its therapeutic potential as an adjunct in nanoparticle-based anticancer strategies. Although the current research used a novel approach that combines apoptosis-related gene expression and molecular docking for the first time to elucidate how green-synthesized nanoparticles are capable of exerting anticancer effects on HepG2 cancer cells, the limitations of the current study lie in the lack of a molecular dynamics assessment and the lack of validation at the protein level. Therefore, future research should concentrate on validating dynamic interactions in vivo and assessing targeted nanoparticle-based delivery systems using specific phytochemicals that modulate apoptosis.

## Figures and Tables

**Table 1:** Molecular docking scores of phytochemicals with apoptotic targets (binding energy in kcal/mol).

Compound	Caspase_3_ (kcal/mol)	Caspase_9_ (kcal/mol)	BAX (kcal/mol)
Aleuritolic acid	−8.4	−8.1	−7.9
Ferulic acid	−7.2	−7.0	−6.8
Flavonoid	−8.0	−7.8	−8.2
Rutin	−9.3	−9.1	−9.0

**Table 2:** Primer sequence used in this study.

Primer		Primer Sequence
Caspase_3_	Forward	TGCCGTGGTACAGAACTGG
	Reverse	CTGGATGAACCAGGAGCCAT
Caspase_9_	Forward	GGATTTGGTGATGTCGAGCAG
	Reverse	AAAGATGTCACTGGGTGTGGG
BAX	Forward	GGGTTGTCGCCCTTTTCTAC
	Reverse	GAGGAGTCTCACCCAACCAC
GAPDH	Forward	GAAGGTGAAGGTCGGAGTC
	Reverse	GAAGATGGTGATGGGATTTC

**Table 3:** Components, volume, and final concentration of PCR master mix.

Component	Volume (µL)
Template cDNA	2 µL
10X PCR Buffer	5 µL
10 mM dNTP mix	1 µL (0.2 Mm each)
25 mM MgCl_2_	3 µL
Forward primer	1.5 µL
Reverse primer	1.5 µL
Taq-Polymerase (5 U/µL)	0.5 µL
Free Water	35.5 µL
Final Volume	50 µL

## Data Availability

The raw data supporting the conclusions of this article will be made available by the authors on request.
